# Modulation of spontaneous locomotor and respiratory drives to hindlimb motoneurons temporally related to sympathetic drives as revealed by Mayer waves

**DOI:** 10.3389/fncir.2015.00001

**Published:** 2015-02-10

**Authors:** Jacob Wienecke, Manuel Enríquez Denton, Katinka Stecina, Peter A. Kirkwood, Hans Hultborn

**Affiliations:** ^1^Department of Neuroscience and Pharmacology, The Panum Institute, University of CopenhagenCopenhagen, Denmark; ^2^Department of Nutrition, Exercise and Sports, The Panum Institute, University of CopenhagenCopenhagen, Denmark; ^3^Sobell Department for Motor Neuroscience and Movement Disorders, University College London Institute of NeurologyLondon, UK; ^4^Universidad del Valle de MéxicoMexico City, Mexico; ^5^Department of Physiology and Pathophysiology, University of ManitobaWinnipeg, MB, Canada

**Keywords:** locomotion, respiration, central pattern generators, Mayer waves, motoneurons, sympathetic drive

## Abstract

In this study we investigated how the networks mediating respiratory and locomotor drives to lumbar motoneurons interact and how this interaction is modulated in relation to periodic variations in blood pressure (Mayer waves). Seven decerebrate cats, under neuromuscular blockade, were used to study central respiratory drive potentials (CRDPs, usually enhanced by added CO_2_) and spontaneously occurring locomotor drive potentials (LDPs) in hindlimb motoneurons, together with hindlimb and phrenic nerve discharges. In four of the cats both drives and their voltage-dependent amplification were absent or modest, but in the other three, one or other of these drives was common and the voltage-dependent amplification was frequently strong. Moreover, in these three cats the blood pressure showed marked periodic variation (Mayer waves), with a slow rate (periods 9–104 s, mean 39 ± 17 SD). Profound modulation, synchronized with the Mayer waves was seen in the occurrence and/or in the amplification of the CRDPs or LDPs. In one animal, where CRDPs were present in most cells and the amplification was strong, the CRDP consistently triggered sustained plateaux at one phase of the Mayer wave cycle. In the other two animals, LDPs were common, and the occurrence of the locomotor drive was gated by the Mayer wave cycle, sometimes in alternation with the respiratory drive. Other interactions between the two drives involved respiration providing leading events, including co-activation of flexors and extensors during post-inspiration or a locomotor drive gated or sometimes entrained by respiration. We conclude that the respiratory drive in hindlimb motoneurons is transmitted via elements of the locomotor central pattern generator. The rapid modulation related to Mayer waves suggests the existence of a more direct and specific descending modulatory control than has previously been demonstrated.

## Introduction

It is a common experience for those who work with decerebrate un-anesthetized preparations that they show a great deal of variability of “state,” both between preparations and during the course of recording sessions. This may be expressed in different ways such as in the susceptibility of motoneurons to show persistent inward currents (PICs) or in the occurrence of a locomotor drive. Here our original aim was to describe the voltage-dependent amplification of CRDPs (Sears, 1964) in lumbar motoneurons in the decerebrate cat, following the demonstration by Kirkwood et al. (2002) that CRDPs may trigger plateau potentials (by PICs) in hindlimb motoneurons, even in barbiturate anesthetized preparations in which PICs are generally depressed. We set out to compare the amplification of the CRDPs with that of other physiologically derived motoneuron inputs, such as locomotor drive potentials (LDPs) (Brownstone et al., 1994) or stretch-evoked Ia excitation (Bennett et al., 1998).

Making direct comparisons between these different drives turned out to be a complicated issue, still unresolved, but during these initial experiments we unexpectedly came across evidence for one of the sources of the variability inherent in these preparations. We observed not only CRDPs, but also periods of spontaneous locomotor activity (with LDPs in the motoneurons) and a number of variable relations between the respiratory and locomotor activities as recorded in both muscle nerves and intracellularly in the motoneurons. Only some of these relations were evident to us during the experiments, which consisted of serial intracellular recordings from motoneurons, together with injections of depolarizing currents intended to reveal voltage-sensitive amplification of the various synaptic drives. On *post hoc* analysis, however, it became evident that many of the motor patterns were related to intermittent increases in blood pressure. These spontaneous increases in blood pressure are called Mayer waves (Mayer, 1876; Montano et al., 1992; Julien, 2006) and are widely believed to be mediated by variation in sympathetic drive, though the origin of that variation is enigmatic (see Julien, 2006 for review). However, the systematic changes in respiratory drive, in activation of locomotor circuits, and in their interaction, provide new clues as to the spinal organization of the networks involved.

There are a large number of publications on how various types of physical activity (walking, running, and jumping) in humans and animals may entrain the respiratory cycles (see Discussion), but here it is the descending respiratory drive to the lumbar motoneurons which seemed to be the dominant rhythm in its inter-relationship with the spinal locomotor network. The relatively rapid modulation of this inter-relationship, and its association with the Mayer waves are the subjects of this communication. Some of the present results were briefly communicated in a congress report (Kirkwood et al., 2005).

## Methods

All surgery and experimental protocols were conducted in accordance with EU regulations (Council Directive 86/609/EEC) and with National Institutes of Health *Guidelines for the Care and Use of Laboratory Animals* (NIH publication no. 86–23, revised 1985), and were approved by the Danish Animal Experimentation Inspectorate. Experiments were performed on seven adult cats of either sex weighing 2.9–3.6 kg. The present experiments were part of longer series of investigations into the respiratory drive recorded in motoneurons in various spinal cord segments, from which observations on phrenic and intercostal motoneurons have recently been published (Enríquez Denton et al., 2012). Many details of the methods can be found there. Here we will give a shorter version, with additional information on the specific arrangements for the hindlimb motoneuron/nerve preparation.

### Anesthesia and decerebration

After the induction of anesthesia with isoflurane–nitrous oxide (2–3% isoflurane, 70% N_2_O, and 30% O_2_), the animals were intubated, and cannulae were inserted into the right carotid artery and the right cephalic and jugular veins for the monitoring of blood pressure and the administration of fluid and drugs. The anesthetic gasses were administered first in an anesthetic chamber, then by a mask and finally via the tracheal cannula, maintained until decerebration was completed (see below).

Six of the seven cats were finally subjected to a mechanical decerebration at precollicular level, including removal of brain tissue rostral to the transection. This was carried out during anesthesia, at the end of the surgical procedure, after the animal had been transferred to the recording frame with a stereotactic head-holder (Gossard et al., 1994). In 5 of the 7 cats we initially performed an anemic decerebration by ligating the basilar and both common carotid arteries, a procedure that has been shown to produce a decerebration that includes all cerebral tissue above the pons and the anterior part of the cerebellum (Pollock and Davis, 1930; Crone et al., 1988; Geertsen et al., 2011). Following the transfer of the cat to the recording frame, the second carotid artery was ligated and the anesthetic removed. Decerebration was verified to be clinically complete by the development of tonic extensor muscle tone, lack of spontaneous movements, and large non-reactive pupils (Crone et al., 1988). However, in 4 of the 5 cats in this series of experiments, stereotyped stepping movements developed after the removal of anesthetics, so the anesthesia was immediately reinstated and the brain was removed rostral to a section at the level of the superior colliculi, just as for the two cats in which the mechanical decerebration was performed without preceding attempt of an anemic decerebration. Following these procedures, neuromuscular transmission was blocked with pancuronium bromide and artificial respiration was initiated (see below).

### Nerve dissection and laminectomy

The following hindlimb nerves (left side) were dissected: posterior biceps and semitendinosus (PBSt), semimembranosus and anterior biceps (SmAB), the gastrocnemius and soleus (GS; in two preparations this nerve was left intact in continuity with the muscles to allow studies on the effect of muscle stretch), the tibial nerve (Tib) and the deep peroneal nerve (DP), which contains the tibial anterior and extensor digitorum nerves. These nerves were cut distally and freed from connective tissue for later mounting on bipolar silver hook electrodes for recording and stimulation (with the exception of the GS nerve in two cats as specified above). For stretch of triceps surae, a hole was drilled through the calcaneus bone, a heavy inelastic nylon cord was tied through the hole, and the bone was cut distally, leaving only a small bone chip attached to the Achilles tendon. The nylon cord was later tied to a muscle puller (310B, Cambridge) (see Bennett et al., 1998). Sinusoidal stretches were applied at 1 Hz. Laminectomy of L4–L6 vertebrae was performed to expose the dorsum of the spinal cord for later intracellular recording from hindlimb motoneurons. Mineral oil pools were fashioned from the loose skin at the laminectomy and hindlimb wound margins. A monopolar recording electrode was placed on the dorsal surface of the lumbar spinal cord to record the afferent incoming volley associated with electrical stimulation of the peripheral nerves. The C5 phrenic nerve was dissected and mounted on buried electrodes for recording efferent discharges. In one animal this recording failed early in the experiment and a T5 external intercostal nerve was prepared as an alternative.

### Maintenance of the preparations

Atropine (0.1 mg kg^−1^, s.c.) and solumedrol (2.5 mg kg^−1^, i.v.) were administered at the beginning of the experiment and a buffer solution (10% glucose and 1.7% NaHCO_3_) was infused continuously (~4.5 ml h^−1^) after cannulation. Neuromuscular blockade was obtained with pancuronium bromide at a dose of 0.6 mg h^−1^ (i.v.) and animals were artificially ventilated with oxygen-enriched air, so as to bring the end-tidal CO_2_ fraction initially to about 4%. A low stroke volume and a high pump rate (typically 1 s^−1^) were employed, so that events related to the central respiratory drive could be distinguished from those due to movement-related afferent input. A bilateral pneumothorax was performed and the end-expiratory pressure maintained at 2–3 cm H_2_O. CO_2_ was then added to the gas mixture to raise the end-tidal level to the value required. The actual value chosen was varied with the aim of controlling the central respiratory drive (end-tidal CO_2_ fractions between 3 and 10%). Rectal temperature was maintained between 37 and 38°C by a servo-controlled warm air flow and a radiant heater. Blood pressure was measured from a cannulated common carotid artery. Mean values were above 80 mm Hg throughout. To assist maintenance of blood pressure Effortil (Etilifrin-hydrochlorid; Boehringer Ingelheim) was administered i.v. in five cats (though in two of them only near the end of the experiment). At the end of the experiment the animals were killed with an overdose of barbiturate.

### Recording

AC-coupled recordings were made of the cord dorsum potentials for incoming afferent volleys and the electroneurograms (ENGs: the phrenic nerve; the external intercostal nerve in one cat; when appropriate, see below, the dissected hindlimb nerves as listed above). Intracellular recordings were DC-coupled, but a high gain output channel high pass filtered at 1 Hz was also included. Intracellular recordings were made from antidromically identified motoneurons, using an Axoclamp 2B amplifier (Axon Instruments) in either standard bridge mode, or in discontinuous current clamp (DCC) mode. Microelectrodes (typical impedance 5 MΩ) were filled with 2 M potassium acetate, and contained the local anesthetic derivative QX-314 (50 mm) to block actions potentials, so as to facilitate the study of the size of EPSPs at different membrane potentials. Note that in several of the records illustrated, a few action potentials survived, showing the QX-314 block to be incomplete at those times. DCC mode was used to allow for more accurate measurements of membrane potential despite changes in electrode resistance with injected current. The DCC cycling rate was typically around 3 kHz with optimal capacitance compensation. Most often slow depolarizing and hyperpolarizing ramps of currents were used (triangular current ramps), but some step changes of constant current levels were also employed. During many of the motoneuron recordings we also recorded efferent discharges from the hindlimb nerves via the same electrodes as used for antidromic identification purposes. This was rarely done in the early experiments, where the focus was on the voltage-dependent amplification of synaptic potentials, but once it was realized that a locomotor drive was sometimes present in the recordings, then these electrodes were switched to their recording mode as soon as antidromic identification had been confirmed. The ENG recordings were done with custom built amplifiers and analog filtering (1–10 kHz) and digitized at a rate of 10 kHz. Full wave rectification and additional filtering was done during analysis so that the onset and the offset of ENG bursts in each nerve were identified by visual inspection of ENG levels crossing a baseline defined by no activity periods. These onset and offset points were used during cycle-based averaging of ENG activity. The data were collected and analyzed with a Canadian software-based QNX-system, developed by the Winnipeg Spinal Cord Research Center to run under a real-time Unix personal computer, usually using separate runs of 200 s duration.

## Results

### Mayer waves and discharges in hindlimb muscle nerves

The initial aim of the experiments was to investigate the occurrence of CRDPs in hindlimb motoneurons of decerebrates such as were previously reported to occur under anesthesia (Kirkwood et al., 2002; Ford and Kirkwood, 2006), concentrating in particular on their voltage dependency. Our monitor nerve discharge was therefore that of the phrenic (or, in one instance, an external intercostal nerve in lieu of the phrenic). In all preparations, CRDPs were common, almost all being of the expiratory decrementing (E_dec_) type, as in Ford and Kirkwood (2006). In 4 out of the 7 experiments, the amplitudes of the CRDPs were relatively small, and often showing some amplification with depolarization, again similar to that previous study, although now with a higher occurrence of plateaux. When tested, no efferent discharges were present in the hindlimb nerves. In contrast, in the remaining three experiments, the respiratory drive in the hindlimb motoneurons was often strong, and additionally at some times during the experiment spontaneous LDPs were also seen. In the first of these experiments, the LDPs were only identified in the recordings *post hoc*, but in the other two experiments, the LDPs were confirmed as such by recording periodic (often flexor-extensor alternating) efferent discharges from the hindlimb nerves. These three animals were also characterized by periodic fluctuations in blood pressure, slower than the respiratory rate, with intervals between the blood pressure maxima ranging from 9 to 104 s, though at any moment during an experiment, the interval could be very variable. The mean interval was assessed in each of the computer runs where the fluctuations could be readily distinguished, the mean of these values being 39 s ± 17 SD (*n* = 71). We are taking these as Mayer waves, despite the frequencies mostly being slower than those classically recognized under this term (see Discussion). Here the Mayer waves appeared variably in the course of the recording sessions, often occurring as isolated phases of increased blood pressure on a steady background (e.g., see later in **Figures 5**, **9**). However, they also could occur, especially when the frequency was higher, as a continuously fluctuating waveform or, occasionally, as periodic dips in blood pressure from a steady high level (e.g., see later **Figure 3**).

Clinically, Mayer waves may be seen in pathological situations (e.g., in hypovolemia), but it should be noted that these three preparations were among those in apparently the best condition. One of them, which provides many of the illustrations here, was specifically noted as such in the experimental protocol, before any recordings had been made. In this animal there had been minimal blood loss during the decerebration procedure and it showed a brisk CO_2_ response in the phrenic nerve, including hypocapnic apnea. An overall characteristic of these three preparations was a minute-to-minute variability of state, as reflected in the patterns of the Mayer waves, CRDPs, LDPs, and nerve discharges. However, a universal observation was that whenever the Mayer wave behavior was marked then either or both of the respiratory and locomotor drives were modulated in relation to the Mayer wave cycle. It is this modulation which is the subject of this report.

**Figure 1:**
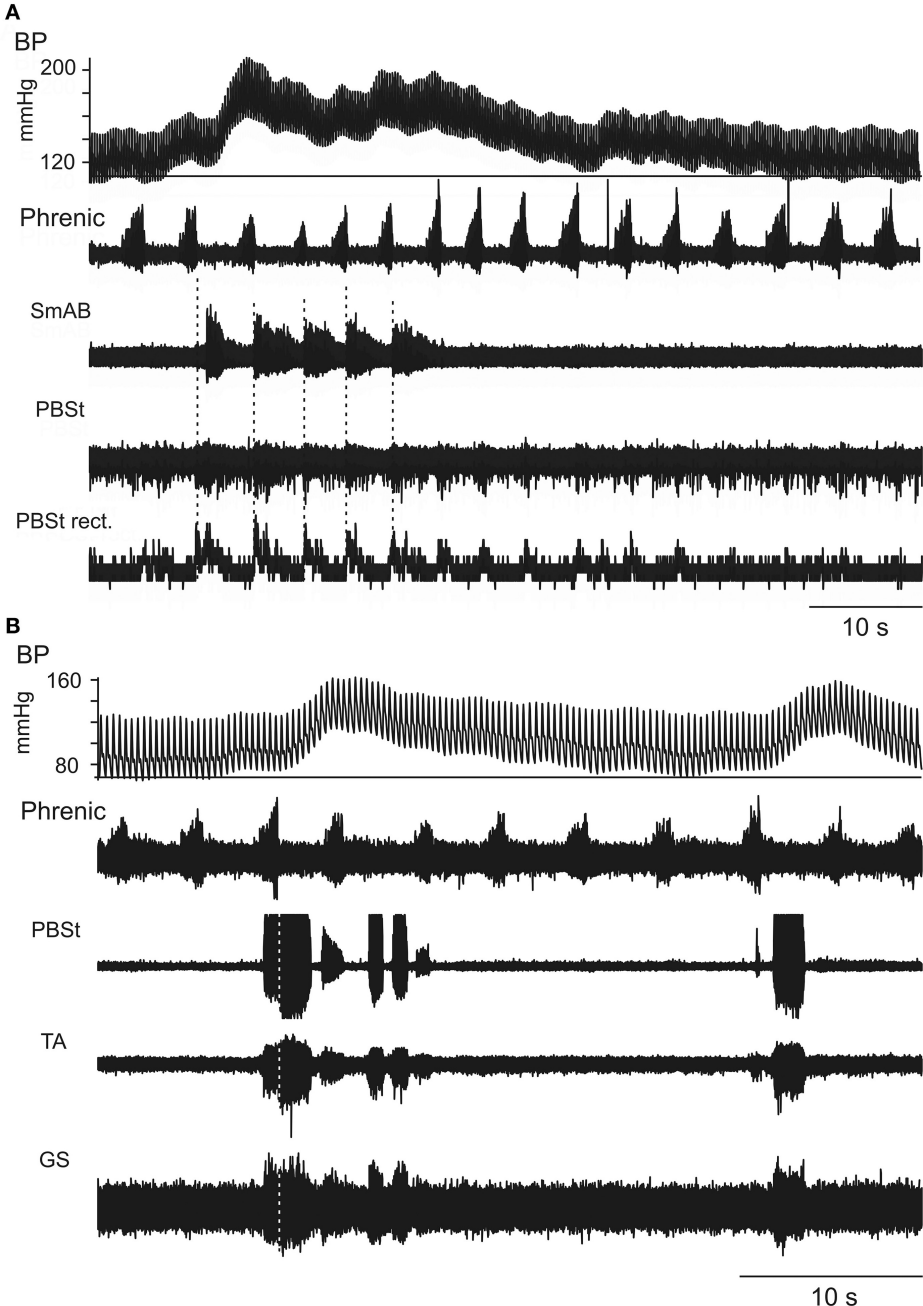
**Two different patterns of coupling between phrenic (inspiratory) discharges and hindlimb muscle nerve discharges**. Appearance of one **(A)** or two **(B)** cycles of Mayer waves, as shown in the blood pressure (BP) traces and the phrenic nerve recordings (second traces). As was common, the phrenic nerve discharges in the respiratory cycle or cycles at or just before the hypertensive phase were more intense than in average respiratory cycles and the discharges during the hypertensive phase were less intense. In **(A)** the initial increase prior to the Mayer wave is difficult to judge as it occurred at the very beginning of the recording, but the less intense discharges during the blood pressure increase are easily seen. The following three traces show **(A)** the raw ENGs from SmAB and PBSt, together with the rectified and integrated version of the PBSt record, **(B)** the raw ENGs from PBSt, TA, and GS. As is typically the case, the intensity of the discharge in these nerves was highest during the hypertensive phase and was higher in the thigh and knee muscles [the PBSt record in **(B)** is saturated], while that in TA and especially in GS were weaker. Note that the typical expiratory decrementing pattern (E_dec_) in **(A)** involved co-activation of flexors and extensors. **(B)** Shows a variant of this, where post-inspiratory bursts were present at the start of the hypertensive phases, but were accompanied by variable bursts during the end of inspiration and later into subsequent respiratory cycles. Vertical dotted lines indicate the end of inspiration.

As illustrated in Figure 1 (and later in **Figure 5**), most often the phrenic nerve discharge in the inspiratory phase just preceding the blood pressure rise was particularly intense while the phrenic nerve discharges in the following respiratory cycles were less intense than in steady state, a modulation with a similar phase difference to that shown by Preiss et al. (1975). Figure 1 includes two different examples of the phrenic modulation, a relatively continuous variation in Figure 1A, and a modest increase of probably only one phrenic burst per Mayer-wave cycle in Figure 1B. Another variation, even closer to the behavior described by Preiss et al. (1975), was one where several respiratory cycles showed an elevated phrenic discharge starting just before the blood pressure rise. An illustration of this was included in the Supplementary data of Enríquez Denton et al. (2012). Figure 1 also illustrates one of the typical patterns of nerve discharges associated with the occurrence of Mayer waves, a co-activation of flexors and extensors. Most often, as in Figure 1A, this generalized nerve discharge occurred in the post-inspiratory phase, briefly after the end of the phrenic nerve discharge (Richter, 1982). However, with the first Mayer wave in Figure 1B the co-activation actually started during late inspiration and it was repeated a few times during the period of elevated blood pressure before falling silent. At the second Mayer wave in Figure 1B there was only a very brief and weak co-activation during inspiration, while the major discharge occurred, as usual, in the post-inspiratory phase. The two examples in Figure 1 were typical of individual Mayer wave cycles in each of those two preparations in the particular states pertaining at the time (see later for a description of state variation). We describe below some of the variations seen in the other states.

**Figure 2:**
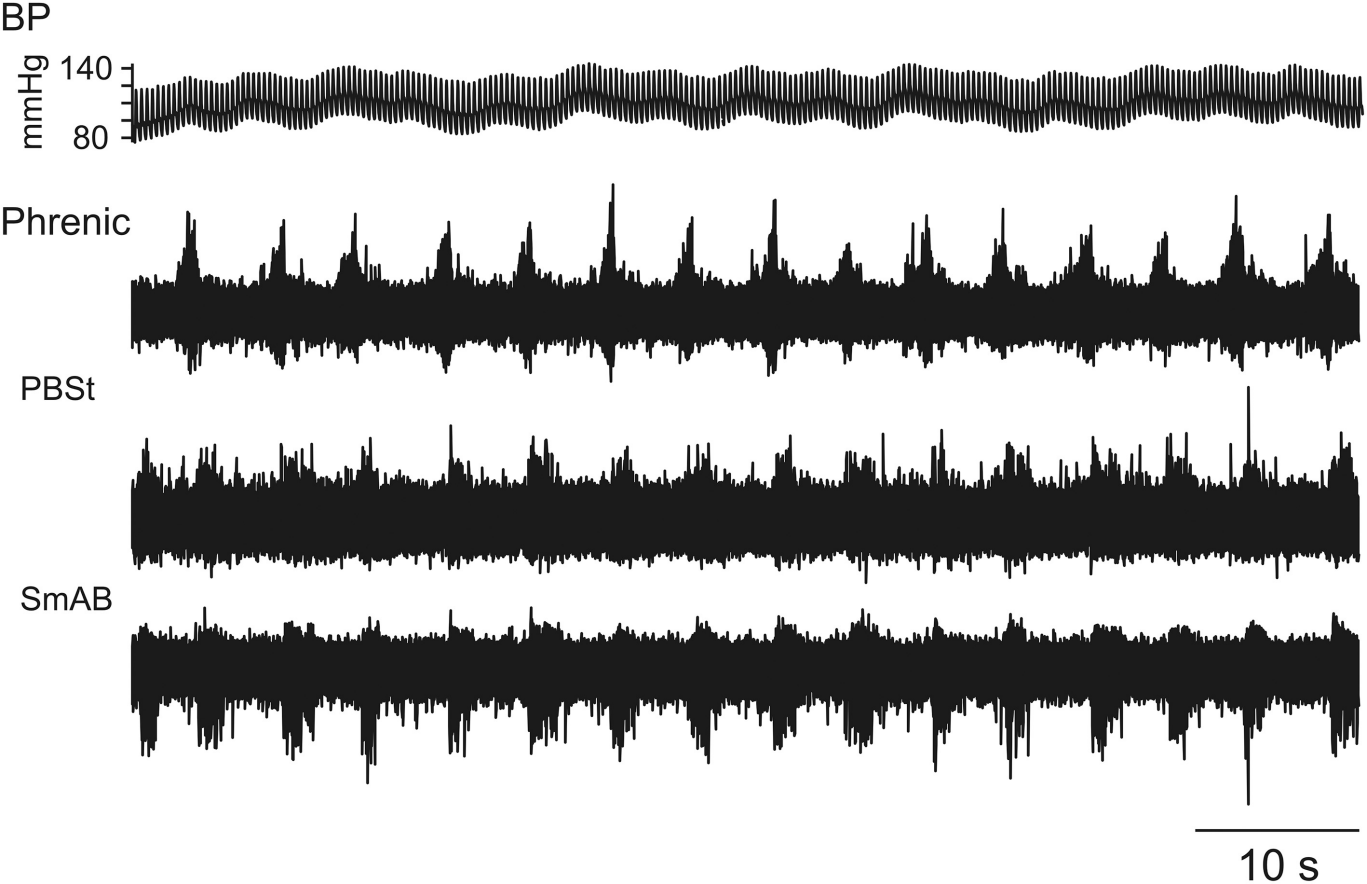
**Maintained post-inspiratory co-activation of hindlimb muscle nerves following every phrenic nerve discharge**. In this case the maintained post-inspiratory co-activation of PBSt and SmAB occurred steadily in the absence of Mayer wave behavior.

In some cases the post-inspiratory co-activation of flexors and extensors occurred for each respiratory cycle for prolonged periods when Mayer waves were weak or absent, as seen in Figure 2.

**Figure 3:**
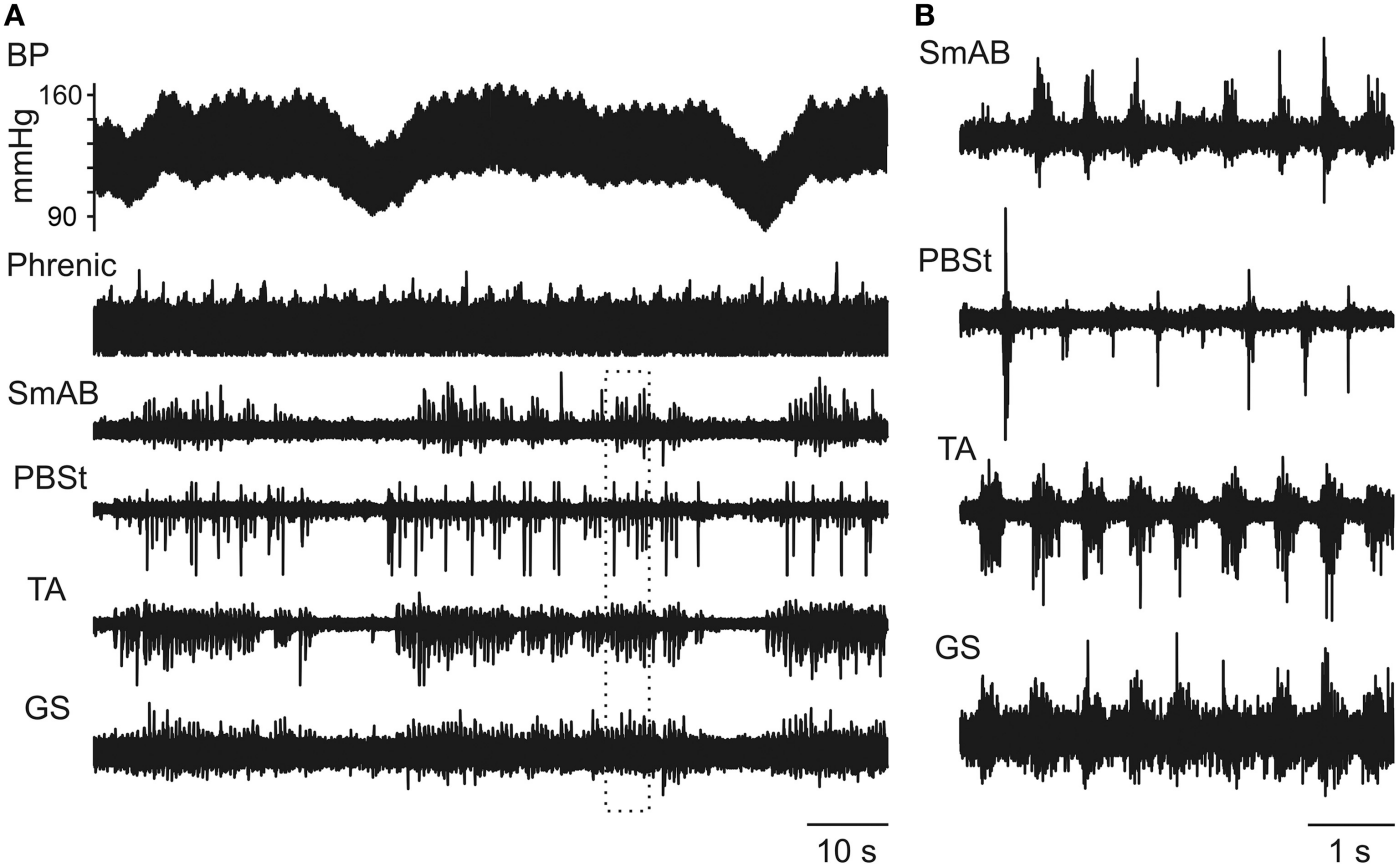
**Spinal locomotor-like activity may be initiated in relation to Mayer waves. (A)** Three Mayer wave cycles are illustrated, where these consisted of relatively short-lasting hypotensive phases from a more steady value. Note that intermittent nerve discharges commence at the BP increase and cease at the BP drop. The alternation between discharges in the different nerves during these periods is seen better in the expanded sweep in **(B)** [from the period marked by the dotted rectangle in **(A)**]. Note that TA and GS were co-activated in this example, though alternating with PBSt. Also note that the intensity of the PBSt (and partly TA) discharges were also modulated according to the respiratory cycle [approximately one respiratory cycle included in **(B)**].

The occurrence of Mayer waves was also frequently related to the initiation of short-lasting episodes of fictive locomotion. One such case is illustrated in Figure 3A in which locomotor-like activity is seen during the high-pressure periods in three Mayer wave cycles. At a faster time base in Figure 3B it is seen that the knee flexors PBSt alternated with the activity in the hip extensor SmAB and ankle extensor GS. However, the antagonist ankle flexor TA nerve activity coincided with the GS activity in this particular example (see further in the Discussion).

### Interference between Mayer wave-related hindlimb nerve activity and spontaneous fictive locomotion

One essential question emerging from the results in the preceding section is related to the identity of the spinal circuits mediating the respiratory-related discharge in the hindlimb muscle nerves. Are they part of the spinal CPG for locomotion or unrelated to that network? In the un-anesthetized decerebrate preparation locomotor-like activity sometimes develops spontaneously (Frigon and Gossard, 2009), as was the case for the three preparations, whose behavior we describe here. In these preparations we noted various examples of perturbations (resetting) and entrainment of the spontaneous locomotor rhythm by the central respiratory activity as monitored by the phrenic nerve discharge, and where the intracellular motoneuron recordings gave us particular insights.

**Figure 4:**
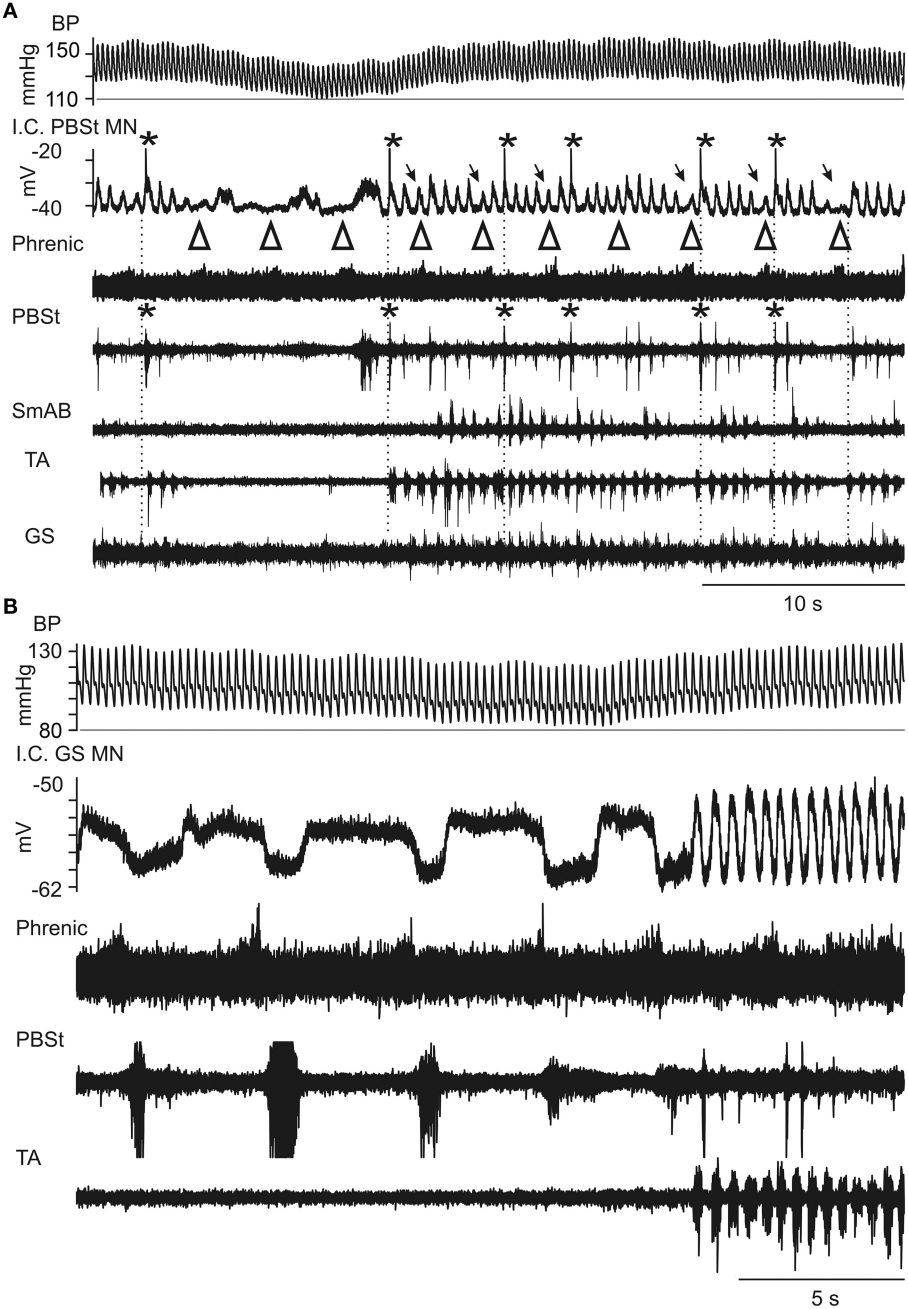
**Expanded records of Mayer wave-initiated locomotor-like activity during intracellular recording from two motoneurons. (A)** From a PBST motoneuron. The largest bursts in the PBSt nerve occurred in the post-inspiratory phase of respiration, corresponding to the largest depolarizations of the LDP in a PBSt motoneuron (asterisks for both). These depolarizations often elicited spikes (truncated). During inspiration, indicated by open arrowheads above the phrenic recording (whose discharge appears weak, most likely a recording problem), there was a decreased amplitude of the LDP (arrows). Between the episodes of locomotor-like activity, the respiratory drive was evident as a CRDP in the PBSt motoneuron, consisting of rather variable depolarization in post-inspiration (arrowheads), and with a corresponding weak discharge, just detectable in the PBSt nerve. The prolonged depolarization visible in the intracellular record following the third arrowhead corresponds to the start of the locomotor episode and probably involved a plateau potential. Same nerve recordings as in Figure 3. **(B)** Similar behavior seen in a recording from the next motoneuron (GS) penetrated in the same preparation. Again, a CRDP is seen in the intracellular recording, in alternation with an LDP, according to the phase of the Mayer-wave. The GS nerve was silent at this time (not shown), but the apparent strong post-inspiratory inhibition in the motoneuron shows the alternation between GS and PBSt for both the CRDP and LDP, similar to that between TA and PBSt during the locomotor episodes. TA was silent during the respiratory episodes, but alternated with PBSt during the locomotor episodes. It is possible that persistent inward current contributed to the depolarizations in this cell, but periods of de- and hyperpolarizations (not shown) indicated that strong phasic inhibition of a tonic background excitation could be sufficient to explain the CRDP/LDP in this instance.

The motoneuron recordings shown in Figure 4 illustrate two aspects of the interaction between the respiratory and locomotor rhythms. In Figure 4A (a PBSt motoneuron) it can be seen firstly that, during the periods when the blood pressure was rising and maintained at a high level (right-hand side of the figure), every discharge in the phrenic nerve (marked by open arrowheads on top of the phrenic nerve ENG) was clearly related to a short interruption of PBSt nerve discharges (partly also seen in the SmAB nerve) together with a striking decrease of the flexor-related LDPs (filled arrows). During such a period, the PBSt discharge was exaggerated in the immediate post-inspiratory phase, as was the depolarization in the motoneuron. Secondly, in the relatively short periods of reduced blood pressure (and also during the decay phase of the preceding Mayer wave), the LDPs were not just interrupted or modulated, but are replaced by a post-inspiratory CRDP, the third cycle of which (immediately before the LDPs commence) appears to involve a plateau potential. A somewhat similar sequence is seen in Figure 4B, where the respiratory and locomotor rhythms again alternated according to the phase of the Mayer wave cycle. This can be seen in the nerve discharges, but is particularly clear in the (GS) motoneuron recording in this instance. At the beginning of the recording (10–20 s, during the decay-phase of the preceding Mayer wave and the period with low blood pressure), the nerve activity was dominated by the respiratory drive then followed by locomotor activity, corresponding to the rising blood pressure. The CRDP in this instance is dominated by a post-inspiratory inhibition (together with a PBSt discharge). This is significant, because it suggests that, at least in some instances when only a single (post-inspiratory) phase of the respiratory drive is evident in the hindlimb discharges, important components of the interneuron circuitry involving reciprocal inhibition appropriate to locomotion may nevertheless be recruited. These examples strongly suggest that the respiratory drive to the motoneurons not only interferes with, but may be mediated by, the spinal locomotor CPG circuits.

**Figure 5:**
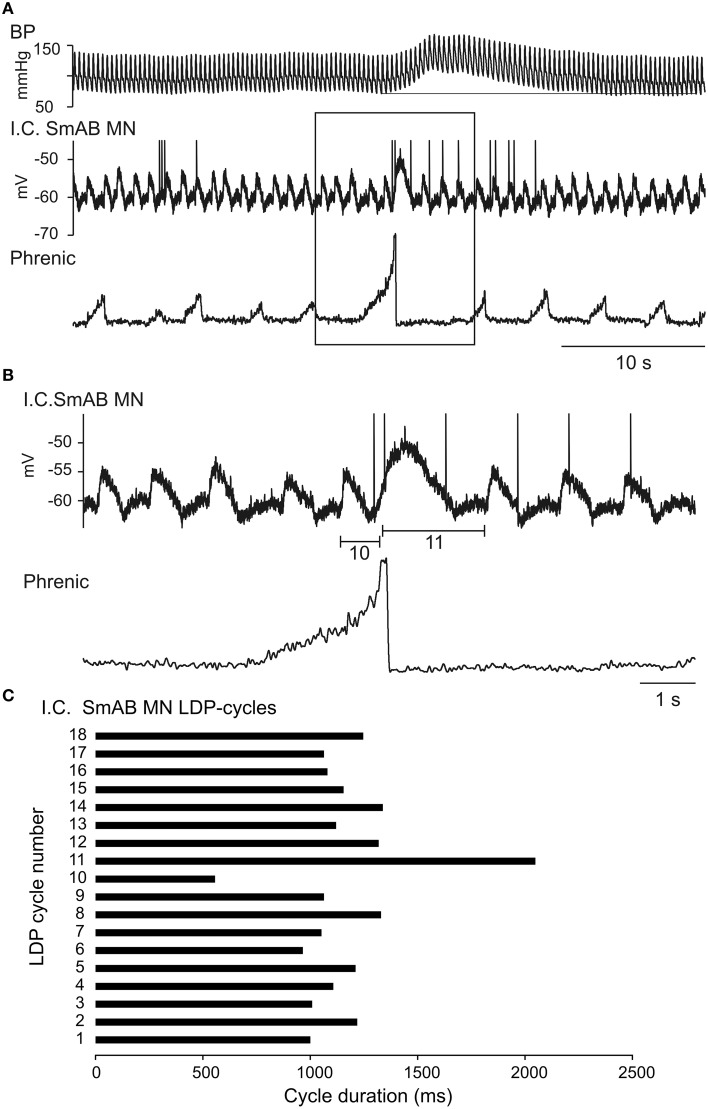
**Demonstration of the interaction between a regular LDP and a single large post-inspiratory depolarization at the beginning of a Mayer wave event**. In **(A)** is shown a rather long-lasting recording of BP, intracellular recording from a SmAB motoneuron and the rectified phrenic nerve ENG with an incident of a large inspiratory discharge. In **(B)** is shown at an expanded time scale the part marked by the rectangle in **(A)**. In **(C)** is shown the cycle duration of the LDPs, the short (no 10) and long (no 11) duration cycles marked in **(B)**.

In Figure 5A there is an example of ongoing locomotor activity (seen as LDPs in the recorded SmAB motoneuron). In the middle of the recorded period there is a large inspiratory discharge (box) followed by a Mayer wave as in Figure 1. The phrenic nerve recording and the intracellular drive potential are shown at a faster time base in Figure 5B. In Figure 5C we show the variation—or rather stability—of the locomotor cycles, with the exception of cycles 10 and 11 marked below the motoneuron recording. It is obvious that in relation to the large phrenic nerve discharge just before the BP increase there is a very short cycle (i.e., the depolarising phase starts earlier than expected) followed by a very long duration for the next cycle. There is resetting of the locomotor rhythm here (the sum of cycles 10 and 11 is longer than any other pair of adjacent cycles), but it is relatively subtle: the timing of the rhythm recovered after a very brief interruption and it is not clear whether the respiratory input really caused an interference with the locomotor CPG rhythm-generator, or whether the modulation coinciding with the Mayer wave event simply slowed the locomotor rhythm and the large depolarisation reflected convergence at motoneuronal level.

**Figure 6:**
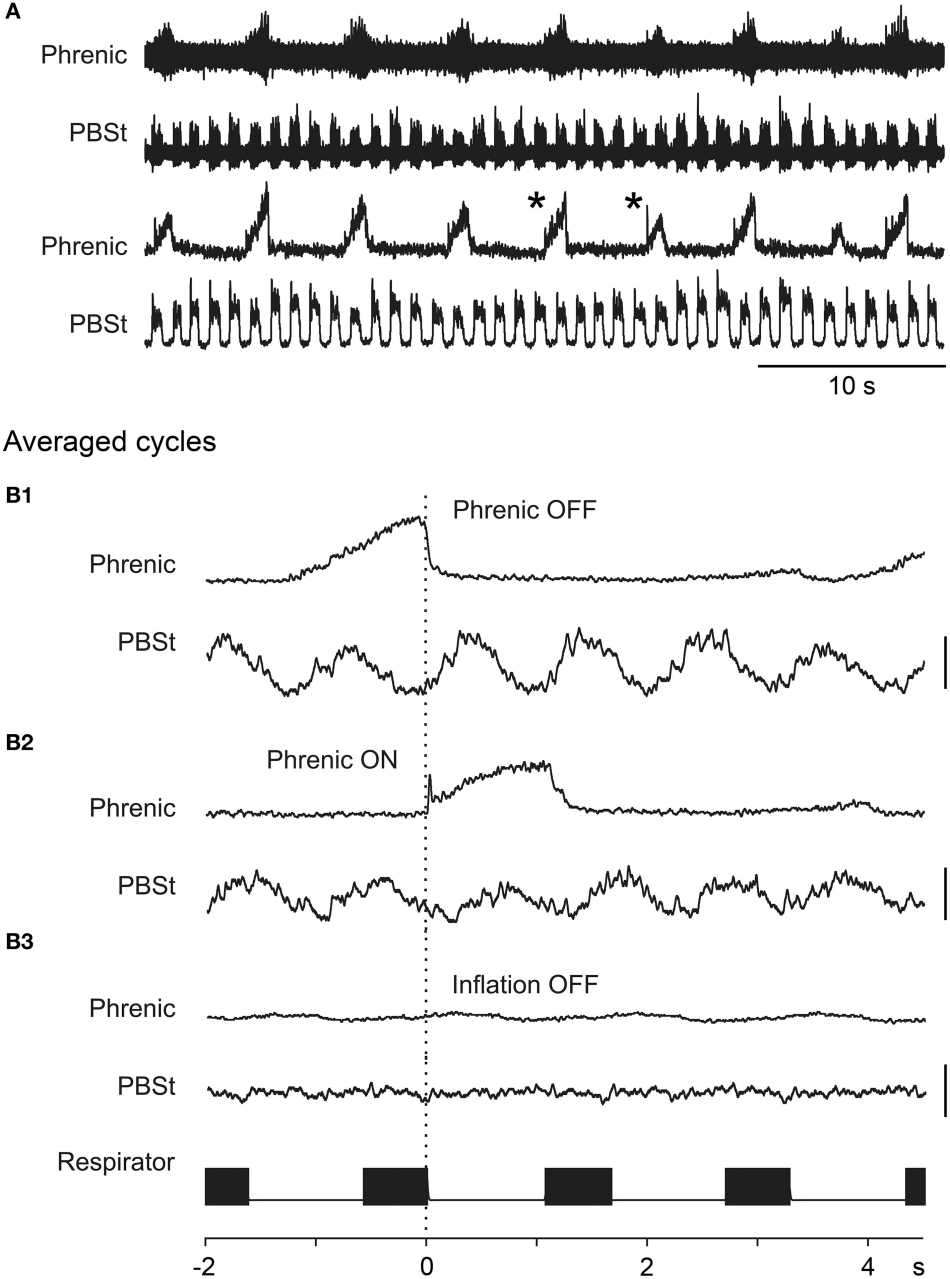
**Entrainment of the locomotor rhythm by the phrenic nerve discharge. (A)** A segment of spontaneous ENG discharges in the phrenic and PBSt nerves. The lower two traces show the integrated rectified versions of the upper two traces. In **(B1–B3)** are shown the discharge in the PBSt nerve where the cycles were averaged in relation to the offset of the phrenic nerve burst **(B1)**, the onset of the phrenic nerve burst **(B2)**, and the offset of the inflation phase of the artificial respiration **(B3)**. The respirator signal was derived from a record of the opening of the inflation valve on this device (filled boxes in respirator trace indicate inflation phases). The averages were derived from a continuous recording, consisting of 17 respiratory cycles; in **(A)** is shown only a part of that recording session. The gains for the averaged traces are constant for **(B1–B3)**, for both the phrenic and the PBSt averages. Calibration bars for the PBSt averages indicates 50% of the average amplitude of individual PBSt integrated rectified bursts. The asterisks in **(A)** indicate two respiratory cycles where a phrenic burst was not followed immediately by a PBSt burst.

The data of Figure 5 come from a long period (about 3 h) in one cat, where a continuous locomotor rhythm was present (see later section). Despite the presence of isolated, relatively large Mayer waves as in Figure 5, there were also periods of minimal Mayer wave activity. We therefore looked carefully at these periods for indications of influence of the respiratory rhythm on the locomotor rhythm independent of Mayer wave occurrence. Figure 6 illustrates one of these periods. In A the raw records are included from a part (33 s) of the 100 s long recording (in all 17 phrenic nerve discharge cycles; only few of them are illustrated in Figure 6A). Even from these raw records it can be seen that there is a tendency for the phrenic discharge to be followed closely by a flexor discharge (illustrated for PBSt), but there were also examples where this was not the case (e.g., the phrenic nerve discharges marked by asterisks in the upper rectified trace in the period illustrated in Figure 6A). The tendency of a post-inspiratory flexor discharge was formally tested for all 17 respiratory cycles. In Figure 6B1 we show the discharges of the PBSt nerve from the recording shown in A, averaged in relation to the offset of the phrenic nerve burst, which demonstrated an apparent entrainment with a post-inspiratory flexor burst, and 4–5 locomotor cycles for each respiratory cycle. Note that the amplitude of the averaged waveform is about 60% of that of the raw integrated rectified PBSt discharge, thus indicating a rather strong phase-coupling. These features (including the same phase relation of the average to the phrenic discharge) were preserved, when this period of 17 cycles was split into two separate periods (7 and 9 cycles, data not shown). The entrainment appears strongest when averaged in relation to the end of the phrenic discharge, most likely because the predominant excitatory event occurred in the post-inspiratory phase and the duration of the phrenic discharge was variable (cf. Richter, 1982). However, the averaged PBSt discharge is clearly visible also when triggered on the onset of the phrenic discharge (Figure 6B2).

The animals were paralyzed and artificially ventilated at a rather high frequency and low volume, with pneumothorax (cf. Methods). Nevertheless, it would have been possible that activation of thoracic, abdominal or vagal mechanoreceptors could have entrained the locomotor (and/or respiratory) rhythm. However, when the PBSt activity was averaged in relation to the pump (offset of inflation) there was absolutely no sign of entrainment (Figure 6B3). Therefore, we conclude that in this case there is a strong entrainment of the locomotor rhythm by the intrinsic respiratory rhythm.

One other such period (20 phrenic cycles) showed the same effect (the same phase), but in five other periods analyzed, this was not really the case. The amplitude of the averaged PBSt ENG was very small suggesting the lack of a strong phase-locking, and thus a lack of evidence for entrainment. Overall, therefore, this long period of continuous locomotor discharge represented a wide spectrum of effects, with examples of a strong coupling and more often an independence between the two rhythm generators.

### Voltage-dependent amplification of LDPs, muscle stretch evoked EPSPs, and CRDPs

**Figure 7:**
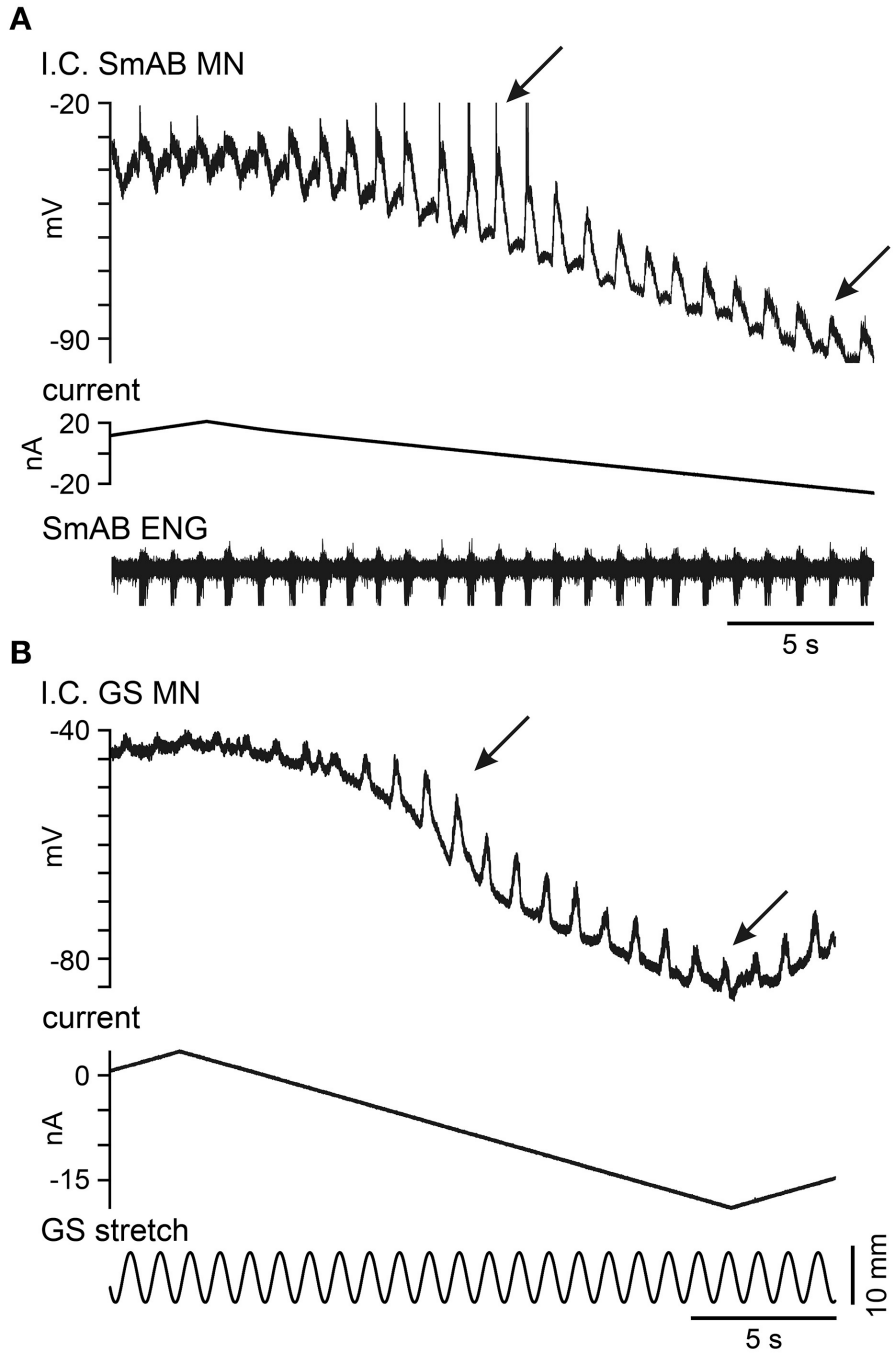
**Voltage dependent amplification of LDPs in a SmAB motoneuron and stretch-evoked Ia excitation in a GS motoneuron. (A)** Recording from a SmAB motoneuron during spontaneous locomotor-like activity. The traces show the LDPs (top), the current injection (middle) and the SmAB ENG (bottom). The voltage dependent amplification of the LDPs (those between the arrowheads) are shown at the descending phase of a triangular current pulse. **(B)** Recordings from a GS motoneuron. The traces show the stretch-evoked EPSPs (top), the current injection (middle) and the sinusoidal muscle stretch of triceps surae (bottom). The arrowheads indicate the region where the amplification was present. At more depolarized levels the amplitude of the EPSP was smaller because of shunting (increased conductance), while the amplitude decreased at more hyperpolarized levels due to the voltage dependent amplification of the EPSP.

A general finding in these experiments was that all of these drive potentials showed a strong voltage-dependent amplification in the voltage region where recruitment of PICs would be expected to contribute to an enhancement of their amplitudes. Such measurements are not always easy to obtain since reciprocal inhibition (through different inhibitory interneurons in between the excitatory periods) also will increase the peak-to-peak amplitude at increasingly depolarized levels. Furthermore, the membrane resistance is reduced at depolarized levels (both by opening of PIC channels and K^+^ channels). Nevertheless, at least qualitative evidence for such amplification was commonly obtained. Figure 7A shows the voltage-dependent amplification of an LDP in a SmAB motoneuron in confirmation of Brownstone et al. (1994). This was demonstrated in 16 out of 16 motoneurons in the present experiments, although a sizeable inhibitory component was suspected in five of these). The decrease of the LDPs is best seen during the hyperpolarizing (descending) phase of the triangular current injection. During the peak of current injection the spikes inactivated and there was an obvious shunting. In Figure 7B (confirming Bennett et al., 1998) there is a similar potential-dependency of the EPSPs evoked by sinusoidal triceps surae stretches (in a GS motoneuron).

**Figure 8:**
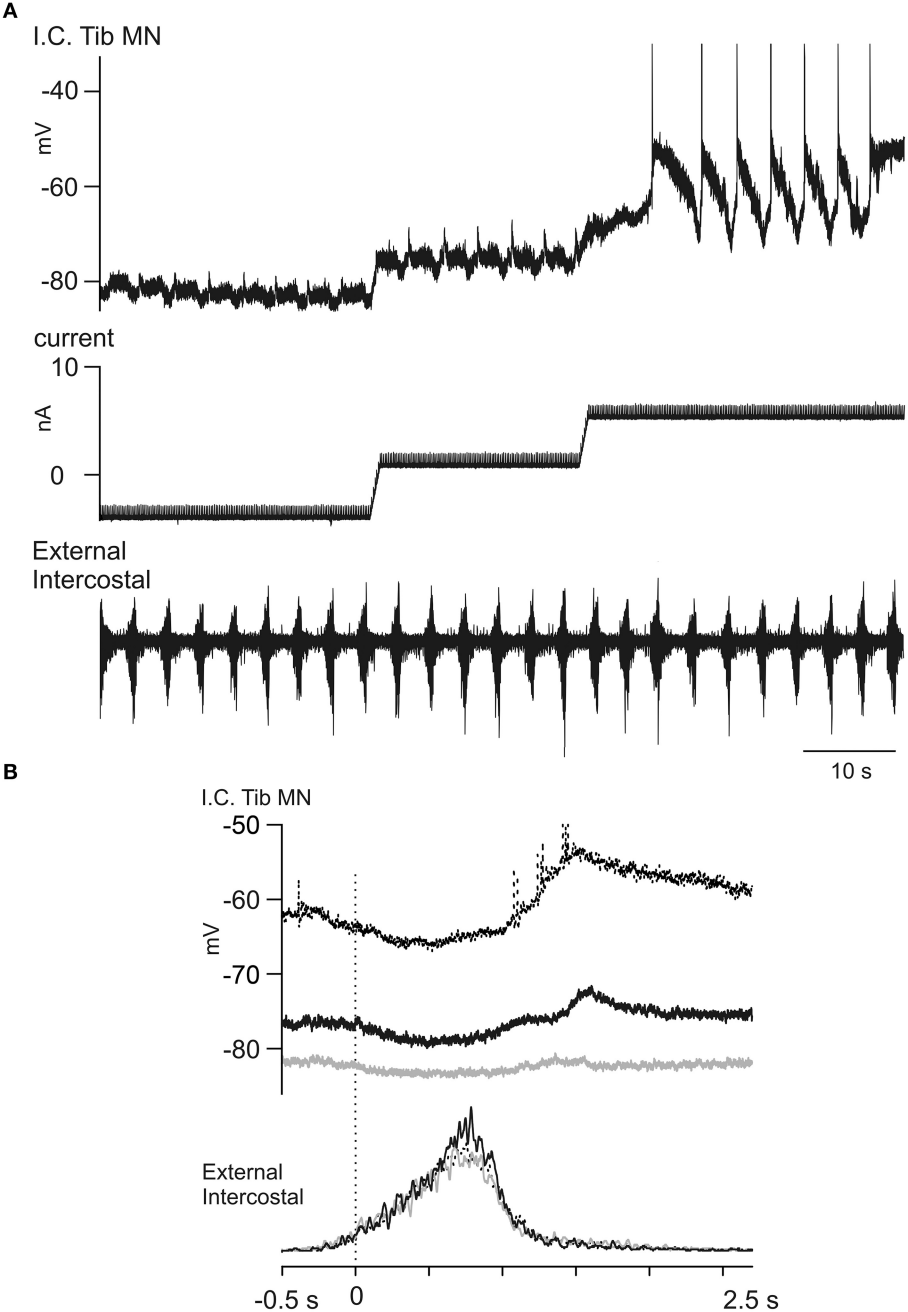
**Voltage dependent amplification of an E_dec_ CRDP. (A)** The traces show a CRDP in a Tib motoneuron (top) during step-wise current injection (middle) and the T5 external intercostal ENG activity (bottom). The CRDP becomes larger at at more depolarized membrane potential levels. **(B)** The averaged CRDPs from **(A)**. The averages were performed separately at the three membrane potential levels (gray, black, and dotted, in increasing order), aligned to the start of inspiration. Note in the average the increased amplitude of the CRDP and the variable times of occurrence for the plateau-like events at +6 nA, which produces a relative attenuation in the averaged signal for this level (*n* = 7, 6, 7, respectively from low to high potential levels). Spikes truncated.

The voltage-dependent amplification of CDRPs (demonstrated in 12 out of 12 motoneurons) was sometimes very dramatic. In Figure 8 we illustrate how a very small E_dec_ CRDP (in a GS motoneuron) was enhanced as the holding potential was increased from −82 to −75 mV and up to −62 mV. At the last level the depolarising phase triggered plateau potentials that usually fell off soon after initiation, and were fully terminated by a subsequent presumed inhibitory phase. These occurred in an all-or nothing fashion. The first two inspiratory discharges after the last depolarizing current step were not followed by plateaux, then the first plateau was not related to the inspiratory discharge, but appeared to be triggered spontaneously. At the very end of the trace the plateau potential was sustained for the whole respiratory cycle. In order to better visualize the potential-dependency we have averaged the intracellular recordings at the three different levels of current injection (−4 nA, +2 nA, and +6 nA) with reference to the beginning of inspiration (Figure 8B). The averages here show first, the presumed inhibitory component during inspiration, increasing, as expected, with depolarization, then, at +2 nA, a small, late depolarization occurred about 0.5 s into expiration. At +6 nA, the plateau-like depolarizations occurred at variable times between 0 and 0.5 s into expiration, as indicated by their initial spike-like components, which are clearly preserved in the average. However, because of this variability, the amplitude of the average plateau is attenuated as compared with the individual events.

### Evidence for changes in intrinsic motoneuron properties in relation to Mayer waves

**Figure 9:**
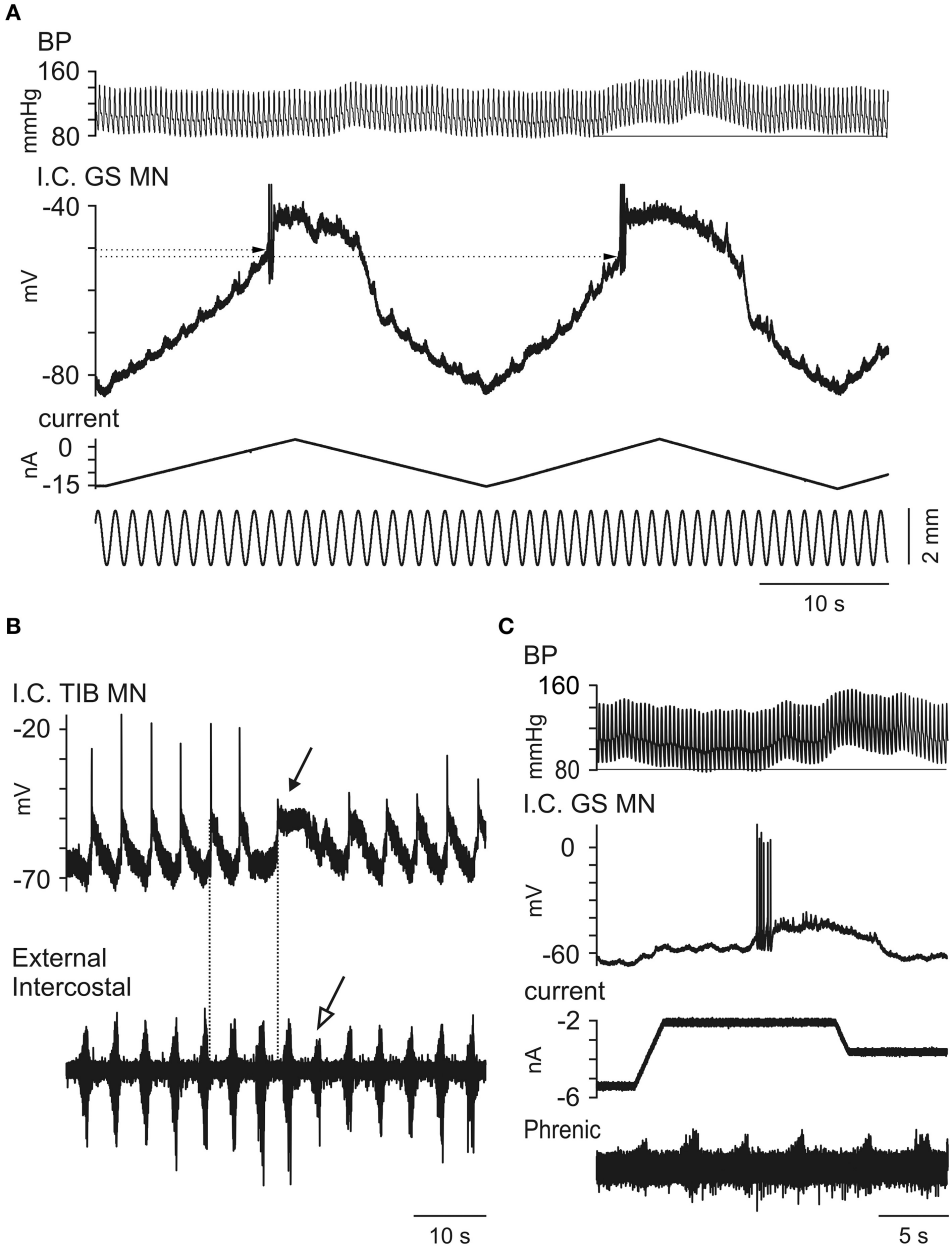
**Observations on changes in intrinsic motoneuronal properties in relation to Mayer waves. (A)**. Recurrent small depolarizations were evoked by small sinusoidal stretches of the triceps surae while triangular current injections were used to initiate plateau potentials. Note that at the beginning of the Mayer wave (at the second triangular increase of current) the threshold for evoking the plateau potential (dotted lines) is lower than during a lower-blood pressure period (i.e., during the first ramp). **(B)** CRDP recorded in a TIB MN. In this case the Mayer wave is inferred by the large external intercostal discharges followed by discharges with decreased amplitude (open arrow). Note the initiation of a plateau potential (filled arrow) even at a time when the CRDP is not expected (compare two dotted lines). **(C)** The traces show the BP, intracellular recording from a GS motoneuron, the injected current and the phrenic ENG. At a constant current injection period, minor potential fluctuations are visible and constant at the beginning of the recorded period, but shortly after the initiation of the Mayer wave one of these triggered both an intense firing and a plateau potential.

There is much evidence for Mayer waves reflecting an increased sympathetic drive at the postganglionic level (see Discussion). However, there is no direct evidence for a simultaneously increased monoaminergic drive to the spinal cord, and we have not aimed to obtain direct evidence for such a drive in the current experiments (see further in Discussion). However, we have looked for signs of changes in intrinsic properties in the motoneurons during Mayer waves that are compatible with an increased monoaminergic drive onto them, promoting PICs and plateau properties. Figure 9A demonstrates how triangular current injections triggered plateau potentials (with a couple of initial spikes). The illustration shows a plateau potential that was initiated and terminated with lower current injection and at a more hyperpolarized membrane potential during the Mayer wave than during the first triangular current injection (no Mayer wave). The longer-lasting plateau during the descending phase during the Mayer wave is particularly striking, but from amplified records we could also confirm that the plateau was initiated at a 2.4 mV more hyperpolarized level (and with 3 nA less depolarizing current injection) than during the first trial before the onset of the Mayer wave. In another illustration, in Figure 9B it is noted that a spontaneous plateau potential was triggered “off-cycle,” toward the end of the phase of increased inspiratory discharges (filled arrow) during the putative Mayer wave (in this case we did not record the blood pressure, but the large intercostal nerve discharges followed by a number of less intensive discharges (marked by an open arrow) at the following respiratory cycles were taken as an indirect indication of the Mayer wave cycle). This same sequence was repeated 5 times in this particular cell. In another motoneuron shown in Figure 9C it may be seen how the small potential fluctuations did not trigger a plateau potential at normal blood pressure levels, but they did so when occurring at the very beginning of a Mayer wave. There were several examples of this kind, but as most of the Mayer waves appeared at irregular intervals a systematic investigation turned out to be difficult. Nevertheless, the present observations are highly suggestive of a neuromodulation that enhances the plateau-properties of motoneurons in relation to Mayer waves.

### Evolution of the general state of each preparation

In the preceding sections we have described a variety of different patterns of motor output. These did not occur at random, but each of the three preparations here showed a systematic change of state during the course of the experiment, though different for each preparation. Each of the observations reported in the preceding sections is only a snapshot, but each is nevertheless representative of longer recordings in that cell, and of the state. The recordings covered periods of 11, 7, and 9 h, respectively for the three preparations, and consisted of individual motoneuron (MN) recordings lasting from about 1 to 40 min. The states of each preparation can be summarized as follows.

#### Cat 1

Initial hindlimb nerve recordings were made in the absence of Mayer waves and showed PBSt discharges during post-Inspiration similar to those in Figure 2 (although that illustration was actually derived from Cat 3). The initial 4 motoneurons yielded an uncertain picture as the phrenic nerve recording gradually failed. By the time an intercostal nerve had been prepared and intracellular recording resumed, Mayer waves were present and small amplitude presumed LDPs, amplified by depolarization, were detectable, sometimes alternating with CRDPs, according to the Mayer wave phase, which itself was clear from the intercostal nerve recording (as in Figure 9B). The amplitude of the Mayer waves was unknown (the blood pressure recording was not acquired by computer in this experiment), but was large enough by the time MN 10 was recorded to be noted in the protocol as an unusually large blood pressure variation being present. The CRDPs then became the dominant feature in the remaining motoneurons (up to MN 22). These CRDPs were strongly amplified by depolarization, involved plateau potentials, and were modulated in phase with the Mayer waves (Figures 8, 9B).

#### Cat 2

Initial nerve hindlimb recordings were made during strong Mayer waves (amplitudes up to 45 mm Hg) with respiratory phased discharges in the nerves strongly modulated in phase with the Mayer waves (Figure 1A). During the early intracellular recordings, Mayer waves were initially absent, then returned (MNs 5, 6), then faded again (MNs 7–9). No LDPs and only very small CRDPS were present. The baseline blood pressure was steady at around 120/80. The phrenic discharge was only modulated when the Mayer wave amplitude was as high as 20 mm Hg. Between MNs 9 and 10 the blood pressure fell, but was restored by a single dose of Effortil (0.5 mg/kg). Following this, a strong locomotor drive became apparent, which persisted during the remaining 4 (relatively long) motoneuron recordings of this experiment. The Mayer wave amplitudes were smaller than in the initial runs (15–20 mm Hg) but nevertheless strong modulation of the motor discharges was apparent in phase with the Mayer waves (Figures 3, 4). The Mayer waves gradually decreased in amplitude, but the modulation of the locomotor and respiratory discharges related to them persisted with amplitudes below 10 mm Hg.

#### Cat 3

This involved the largest number of motoneuron recordings (MN1–MN33). For the first 14 motoneuron recordings, only low amplitude Mayer waves were present, only low amplitude CRDPs and no LDPs. A weak respiratory-phased discharge was present in SmAB nerve in the one recording tested in this period. Modulation of the CRDPs and of the phrenic discharges in relation to the Mayer waves was only seen toward the latter part of this period (MNs 9–14) and only for larger amplitude Mayer waves (10–15 mm Hg). Then, for MNs 16–26, continuous LDPs were present, together with continuous locomotor discharges in the hindlimb nerves, as in Figures 5, 6. Mayer waves were only sometimes present, generally of low amplitude (≤15 mm Hg), and these were not accompanied by any obvious modulation of the motor output. However, occasional isolated large amplitude Mayer wave events (up to 40 mm Hg) also occurred, accompanied by resetting of the locomotor rhythm with regard to the respiratory cycle (Figure 5). For MNs 27–31, the Mayer waves became stronger and more regular, being accompanied first by a continuous respiratory drive in the hindlimb nerves in the periods between medium-amplitude Mayer wave events (Figure 2), then by the strongly modulated short (locomotor?) repetitive bursts synchronized to respiration and to large amplitude (45 mm Hg) Mayer waves (Figure 1B). For the last two motoneuron recordings, the Mayer wave amplitude decreased, as did the modulation of the locomotor discharges.

Overall, therefore, although not designed for this purpose, the structure of the experiments, which consisted of repeated measurements over many hours, revealed two or three different time scales of spontaneous modulatory variation. The first one, varying over hours to minutes, seemed to consist of the extent to which locomotor drives were present at all, a respiratory drive being present throughout (as it was in the four experiments not showing Mayer waves). The second one, varying over minutes to seconds determined, in phase with the Mayer waves, which of the two rhythms was transmitted to the motoneurones. Finally, the Mayer waves themselves waxed and waned independently of the locomotor drive, but over a similar time scale.

## Discussion

### Relations between the neuronal networks of respiration and locomotion at lumbar level

In this report we have demonstrated that at spinal level there are intricate interrelations between the networks mediating a respiratory control of lower limb muscle activity and locomotor activity. In the decerebrate, unanesthetized and paralyzed preparation which we have used, the activation of these interneuronal circuits—and their interrelation—varied substantially with the spontaneous occurrence of periods of increased blood pressure, i.e., the so called Mayer waves. During these periods the intrinsic behavior of motoneuron properties changed as might be expected if an increased monoaminergic drive to the spinal cord occurred in phase with the presumed sympathetic activity during the Mayer waves (cf. the examples in Figure 9, see below). It is well-established that monoamines are important transmitters in activating the spinal network underlying locomotor activity, the spinal locomotor CPG, as first demonstrated for the cat (administration of l-DOPA: Jankowska et al., 1967a,b; Grillner and Zangger, 1979, or the α_2_ agonist clonidine, Forssberg and Grillner, 1973; Barbeau et al., 1987; Kiehn et al., 1992; Chau et al., 1998; Noga et al., 2006, 2007). In addition monoamines (NA; DA and 5-HT) have been used to activate spinal locomotion in the rabbit (Viala et al., 1979) and the marmoset monkey (Fedirchuk et al., 1998) as well as in adult rats (Iles and Nicolopoulos-Stounaras, 1996) and mice (Meehan et al., 2012). Monoamines, often in combination with NMDA receptor agonists, also activate the spinal CPG in the neonatal rat and mouse, (reviews; Schmidt and Jordan, 2000; Clarac et al., 2004; Miles and Sillar, 2011). Readers should also consult other articles in this Research Topic.

In general, the locomotor pattern evoked by the pharmacological cocktail is characterized by alternating flexor and extensor bursts, and by alternation between the two hindlimbs. However, at the start of the bursting activity it has been frequently noted that flexors and extensors were initially co-activated, as were sometimes the two limbs, and that the regularly alternating pattern only subsequently developed during the course of the recording session (Fedirchuk et al., 1998 in the marmoset monkey; Meehan et al., 2012 in the mouse; and more infrequently in the cat preparation, unpublished observation from Hultborn's laboratory). These observations are relevant for the interpretation of the pattern of co-activation of flexors and extensors in the post-inspiratory phase (Figures 1, 2). One interpretation would be that the interneurons mediating such co-contraction are different from the locomotor circuits, but an alternative explanation would be that the respiratory drive is activating parts of the locomotor network in a functional configuration which causes a co-activation. As support for the latter explanation the prolonged locomotor periods in Figures 3, 4 actually display a co-activation of the ankle extensors (GS) and flexors (TA), although flexors and extensors otherwise were alternating. Even more convincing evidence for the respiratory drive working onto—and at least partly through—the locomotor circuits are the inhibitory components of the CRDP in Figure 4B, together with the possible resetting (Figure 5) and certain entrainment (Figure 6) of the locomotor rhythm by the respiratory one.

A correlation between actual motor activity and respiration has long been recognized, and is of course related to increased demand for oxygen and removal of CO_2_. The regulation of the increased ventilation is complex and involves both feed-forward and feed-back mechanisms. It has been studied in humans (Krogh and Lindhard, 1913; Asmussen, 1983; Haouzi, 2006), as well as during the initiation of locomotion in the decerebrate cat (DiMarco et al., 1983). In relation to locomotion, several groups have established an entrainment of the respiratory rhythm by the locomotor activity (Kawahara et al., 1989), which to a large extent seems to originate from limb proprioceptive inputs (Potts et al., 2005; Giraudin et al., 2012), but there is also evidence for a central entrainment of the respiratory pattern by the spinal locomotor circuits (Perségol et al., 1988; Le Gal et al., 2014). The interactions between these two CPGs, the medullary respiratory one and the spinal locomotor one have been described at length in a series of papers from Viala and her colleagues, including also the likely cervical spinal respiratory oscillator, which is not of concern here. In nearly all of these papers the focus is on the dominance of the locomotor over the respiratory CPG, though in two publications from this group (Viala, 1986; Perségol et al., 1988), the authors allowed for the reverse to occur. In these papers (both including fictive activations) the authors describe how the dominance of one rhythm over the other can be varied. The present observations, which show an entrainment of the locomotor circuits by the respiratory rhythm, are closest to their data in the circumstances of a raised level of CO_2_, which was also the circumstance of most of our observations. However, their preparations were different from ours: rabbits with, for the critical observations, a very caudal decerebration and locomotion induced pharmacologically. We suggest the dominance of the respiratory over the locomotor CPG was even stronger for our observations, because the high level of CO_2_ was combined with only spontaneous locomotion. Some of our recordings, where a locomotor pattern alternated with a respiratory one were also remarkably similar to one previous publication in the cat (Figure 5B in Romaniuk et al., 1994). It may be proposed that by stimulating within the more caudal part of the “locomotor strip” (in the medulla) Romaniuk and his colleagues also gave only a relatively weak drive for locomotion. The only difference between their result and ours is that their forelimb nerve discharge showed an inspiratory rather than post-inspiratory pattern when the locomotor pattern did not prevail. However, this is quite compatible with previous observations of respiratory drives recorded intracellularly in forelimb (Enríquez Denton et al., 2012) or hindlimb (Ford and Kirkwood, 2006) motoneurons. By showing the linkage between the two CPGs our present observations also help to explain the many various earlier observations of respiratory influences on limb functions. Meyer-Lohman (1974) can be consulted for an early description of post-inspiratory effects in hindlimb motoneurons, and for even earlier references of respiratory effects.

The question of what function is served by these interactions remains. In addition to variable interactions between the central rhythm generators for spinal locomotor and respiratory control in the adult preparations, the coactivation has also been described in the fetal (Greer et al., 1992) and in the neonatal rat preparations (Morin and Viala, 2002). The coactivation of these CPGs and their variable coupling likely represents an “open-loop” operation of these networks with reduced “functional” control, as both feed-forward and feed-back mechanisms are depressed in the preparations used for studying them. In other words, the inter/descending/propriospinal-neuron networks coupling these two systems are hard-wired in the spinal-brainstem circuits very early during development, and they become very much under the control of sensory feedback and feed-forward signaling from the brain as the animals mature. Under such control, the role of the interactions could be many and varied (see Discussion in Schomburg et al., 2003).

For interactions in the direction of locomotion to respiration, the function is the most obvious, and has been much discussed in terms of respiratory efficiency, or for minimum work in general, especially in situations such as galloping or, for birds, flying (Boggs, 2002). In the opposite direction, as suggested here, the function is less obvious. In this context, it should be remembered that the respiratory CPG, or the neurons within it, can be readily configured for other functions, which include vomiting, coughing, defecation, perhaps even mating (for references see Kirkwood and Ford, 2004). Thus, the influences we are reporting from decerebrate preparations may reflect the operation of connections evolved for motor acts other than respiration, and where the roles of the limbs may be more essential. These roles may also be different for the fore- and the hindlimbs. For the latter the connections may serve the functions just suggested, for the former they may serve accessory inspiratory actions. A large number of possible functions (largely still speculative) for the post-inspiratory (or E_dec_) pattern of excitation in hindlimb motoneurons were discussed by Ford and Kirkwood (2006).

### Do Mayer waves reflect an increased descending monoaminergic drive to the spinal cord?

First we will summarize some facts and questions about the so-called Mayer waves. Slow oscillations (6–9 cycles/min) in arterial pressure were described by Mayer in 1876 in anesthetized rabbits (Mayer, 1876). The review by Julien (2006) describes that the dominant frequency varies between species (~0.1 Hz in humans; ~0.3 Hz in rabbits and ~0.4 Hz in rats and mice), and also that the value depends on the experimental circumstances such as anesthesia, body position and circulatory conditions. In experimental animals and in humans, simultaneous recordings of low frequency increases in arterial blood pressure (at much slower frequency than the respiratory movements themselves) and efferent sympathetic activity have revealed a strong correlation (see review by Julien, 2006). Nevertheless, the origin of these oscillations has been the subject of much experimental work and discussion, mainly focusing on two possible explanations which are not mutually exclusive; (1) the pacemaker theory assuming a central oscillator (in the brain stem) for the genesis of Mayer waves, and (2) the reflex theory emphasizing the baroreflex loop. A large number of experimental studies (reviewed by Julien, 2006) support both hypotheses. In animal experiments the baroreceptor loop can be easily opened either by surgical denervation or by pharmacological means, and this intervention certainly reduces the Mayer wave activity. On the other hand there are observations on slow waves of sympathetic nerve activity even when the blood pressure is clamped and the baroreceptor loop opened, thus emphasizing the presence of a central pacemaker (Preiss and Polosa, 1974; Preiss et al., 1975). As pointed out by Malpas (2002), evidence such as baroreceptor denervation reducing Mayer wave activity is very weak as to the baroreceptor role in an oscillating feedback loop, it only shows that these receptors provide a facilitatory input. However, experiments which show that the activity survives the opening of such a loop, such as those in Preiss and Polosa (1974), provide very strong evidence that Mayer waves can be generated purely centrally. Modern reviewers are dismissive of this evidence, because the Mayer wave frequency in those experiments was much lower and the amplitudes of the pressure variations were much higher than those usually now considered as Mayer waves, and also because Preiss and Polosa (1974) induced these waves by severe hypovolemia. In terms of amplitude and frequency of the blood pressure variation, our experiments are close to those of Preiss and Polosa, though, as mentioned in the first section of the Results, they did not need a hypovolemic stimulus. We also have independent evidence for a central origin of the Mayer waves here, in that the motor actions were fictive and, in the majority of cases where the Mayer waves appeared as isolated increases from a flat baseline, the first event was an increase in the phrenic discharge, before the rise in blood pressure occurred, similar to the phase difference reported by Preiss et al. (1975), also for fictive respiration in decerebrate preparations.

A different question is related to the identity of the descending pathways mediating the activation of the preganglionic sympathetic neurons. It has been demonstrated that neurons of the caudal raphe nuclei project to the intermediolateral cell column (Bacon et al., 1990), but investigations on the serotonergic actions on the sympathetic activity has been dogged by methodological problems (cf. review by Lovick, 1997), although there is evidence for a 5-HT-2-mediated excitation (Orer et al., 1996). There is also evidence for Mayer-wave related modulation in several of the caudal raphe neurons. Bruinstroop et al. (2012) have more recently documented a strong projection from the locus coeruleus onto the thoracic intermediolateral cell column where most of the cells are excited by α_1_ receptors (Lewis and Coote, 1990a,b; see also Samuels and Szabadi, 2008). There is, however, no direct evidence for locus coeruleus neurons to be activated in relation to the Mayer waves.

Even though Mayer waves are correlated to efferent sympathetic activity there is so far no direct evidence for an increased descending monoaminergic drive to the spinal cord during these waves. It would be plausible that the brainstem activity during the Mayer waves is activating the preganglionic neurons in the intermediolateral cell column via descending glutamatergic pathways, which may also activate the spinal locomotor circuits. It is well-established that monoaminergic innervation of the spinal cord affect the intrinsic properties of the motoneurons, enhancing their PICs and thus their plateau properties (see e.g., Hounsgaard et al., 1988; Hounsgaard and Kiehn, 1989 for original articles and several subsequent reviews: Powers and Binder, 2001; Heckman et al., 2003, 2005, 2009; Hultborn et al., 2004; ElBasiouny et al., 2010; Zhang et al., 2012). However, the facilitation of the “plateau properties” in the motoneurons could possibly also be explained by activation of glutamatergic NMDA receptors (cf. Delgado-Lezama et al., 1997; Guertin and Hounsgaard, 1998; Enríquez Denton et al., 2012). It thus now seems warranted to directly investigate the monoaminergic levels during Mayer waves e.g., with voltametric techniques (cf. Hentall et al., 2006).

Further investigation is also warranted as to whether the behavior reported here represents a physiological mechanism or an epiphenomenon. The new observation here of the modulation of CPG interaction, and probably also of the motoneuron intrinsic properties, by a fast-acting descending, naturally occurring drive, is certainly a mechanism which could be physiologically useful. This drive is related, via the Mayer-waves to the control of blood pressure. In broad terms, control of respiration and blood pressure are regarded by Holstege as part of the “emotional motor system,” which he describes as that involved in survival behaviors (Holstege, 1991). In this context, an association with limb motor control should be of no surprise. One particular aspect of that, related to plateau potentials evoked by the respiratory drive, has already been suggested (Kirkwood and Ford, 2004), but there could be many others. In the decerebrate preparation without any specific stimulation, only some features of that association are likely to be detectable.

## Author contributions

Conception and design of study: Jacob Wienecke, Manuel Enríquez Denton, Peter A. Kirkwood, Hans Hultborn. Acquisition, analysis and/or interpretation of data: Jacob Wienecke, Manuel Enríquez Denton, Katinka Stecina, Peter A. Kirkwood, Hans Hultborn. Drafting the ms.: Katinka Stecina, Peter A. Kirkwood, Hans Hultborn. Critical review of the ms. and its final approval: Jacob Wienecke, Manuel Enríquez Denton, Katinka Stecina, Peter A. Kirkwood, Hans Hultborn.

### Conflict of interest statement

The authors declare that the research was conducted in the absence of any commercial or financial relationships that could be construed as a potential conflict of interest.
